# Evolutionary stasis of a heritable morphological trait in a wild fish population despite apparent directional selection

**DOI:** 10.1002/ece3.5274

**Published:** 2019-06-11

**Authors:** Ronan James O'Sullivan, Tutku Aykanat, Susan E. Johnston, Adam Kane, Russell Poole, Ger Rogan, Paulo A. Prodöhl, Craig R. Primmer, Philip McGinnity, Thomas Eric Reed

**Affiliations:** ^1^ School of Biological, Earth & Environmental Sciences University College Cork Cork Ireland; ^2^ Environmental Research Institute University College Cork Cork Ireland; ^3^ Organismal and Evolutionary Biology Research Program, Faculty of Biological and Environmental Sciences University of Helsinki Helsinki Finland; ^4^ Institute of Evolutionary Biology, School of Biological Sciences University of Edinburgh Edinburgh UK; ^5^ School of Biology and Environmental Science and Earth Institute University College Dublin Dublin Ireland; ^6^ Marine Institute, Furnace Newport Mayo Ireland; ^7^ Institute for Global Food Security, School of Biological Sciences, Medical Biology Centre Queen's University Belfast Belfast UK

**Keywords:** Atlantic salmon, Breeder's equation, pedigree, phenotypic selection, secondary theorem of selection

## Abstract

Comparing observed versus theoretically expected evolutionary responses is important for our understanding of the evolutionary process, and for assessing how species may cope with anthropogenic change. Here, we document directional selection for larger female size in Atlantic salmon, using pedigree‐derived estimates of lifetime reproductive success as a fitness measure. We show the trait is heritable and, thus, capable of responding to selection. The Breeder's Equation, which predicts microevolution as the product of phenotypic selection and heritability, predicted evolution of larger size. This was at odds, however, with the observed lack of either phenotypic or genetic temporal trends in body size, a so‐called “paradox of stasis.” To investigate this paradox, we estimated the additive genetic covariance between trait and fitness, which provides a prediction of evolutionary change according to Robertson's secondary theorem of selection (STS) that is unbiased by missing variables. The STS prediction was consistent with the observed stasis. Decomposition of phenotypic selection gradients into genetic and environmental components revealed a potential upward bias, implying unmeasured factors that covary with trait and fitness. These results showcase the power of pedigreed, wild population studies—which have largely been limited to birds and mammals—to study evolutionary processes on contemporary timescales.

## 1 INTRODUCTION

The process of adaptive evolution can be split conceptually into inheritance on the one hand, and phenotypic selection on the other hand, that is, the effect of phenotype on relative fitness. Selection can be thought of as the “bridge” between ecology and evolution (Hendry, 2016), and indeed, changing patterns of selection on functional traits lie at the heart of many applied eco–evolutionary problefms (Alberti, 2015; Fugère & Hendry, 2018; Hanski, 2012; Kinnison & Hairston, 2007; Smallegange & Coulson, 2013). A better understanding of which traits are under selection, the form such selection takes (stabilizing, disruptive, fluctuating, directional), and the extent to which genetic constraints influence actual responses to selection is required to obtain deeper insights into the evolutionary process.

The theoretical groundwork for the study of phenotypic selection in the wild was in place by the 1980s (Arnold & Wade, 1984; Lande, 1980; Lande & Arnold, 1983; Price, 1970), and since then a wealth of empirical studies has reported estimates of selection differentials or gradients in natural populations (Hoekstra et al., 2001; Kingsolver et al., 2001; Kingsolver & Pfenning, 2007; Siepielski, DiBattista, & Carlson, 2009; Siepielski et al., 2013). At the same time, increasing numbers of studies using powerful, flexible statistical approaches such as the “animal model” (Kruuk, 2004; Wilson et al., 2010) report estimates of key quantitative genetic parameters that influence microevolutionary responses. A general finding is that abundant genetic variation exists in natural populations for traits under selection (Lynch & Walsh, 1998; Mousseau & Roff, 1987), and hence, it would be expected that adaptive evolutionary responses should be commonly observed. However, among those studies that have estimated actual microevolutionary trends, a majority have found a lack of observed response to selection, despite evidence for directional selection and heritability; the so‐called “paradox of stasis” (Kruuk, Slate, & Wilson, 2008; Merila, Kruuk, & Sheldon, 2001; Pujol et al., 2018; Stinchcombe, Simonsen, & Blows, 2014).

Accurately estimating the form, direction, strength of selection, and predicting a trait's evolutionary response also has practical applications with the potential to inform management policy for exploited species experiencing harvest‐induced selection (i.e., Allendorf & Hard, 2009), or conservation policy for populations where in situ adaptation to anthropogenic change may be the sole route to persistence (Martins, Kruuk, Llewelyn, Moritz, & Philips, 2018; Visser, 2008). Explanations for mismatches between observed and expected responses to selection, including the special case of evolutionary stasis, can be grouped into biological versus statistical (Pujol et al., 2018). On the biological side, inaccurate microevolutionary predictions can result by failing to account for various phenomena such as age structure, indirect genetic effects, genotype‐by‐environment interactions, fluctuating selection at unmeasured times and/or places, and genetic correlations between the focal trait and unmeasured traits also under selection (Etterson & Shaw, 2001; Morrissey et al., 2012). Statistical explanations invoke biased and/or imprecise estimates of quantitative genetic parameters, for example, failure to account for environmental sources of phenotypic resemblance among relatives (Kruuk & Hadfield, 2007), or bias in phenotypic selection estimates caused by covariance between some unmeasured variable with both the focal trait and fitness (Fisher, 1958; Hadfield, 2008; Morrissey, Kruuk, & Wilson, 2010; Reed, Gienapp, & Visser, 2016; Stinchcombe et al., 2014).

Here, we explore patterns of phenotypic selection, inheritance, and evolution of body size in a wild, pedigreed population of Atlantic salmon (*Salmo salar* Linnaeus, 1758). Body size is a key phenotypic trait generally theorized to be under natural and/or sexual selection. Empirical studies of wild animal populations have found a range of patterns, including positive directional (Boag & Grant, 1981; Brown & Brown, 1998; Husby, Hille, & Visser, 2011; Kruuk, Merilä, & Sheldon, 2001; Milner, Albon, Illius, Pemberton, & Clutton‐Brock, 1999; Schluter & Smith, 1986), negative directional (Bonnet, Wandeler, Camenisch, & Postma", 2017; Price, Grant, Lisle Gibbs, & Boag", 1984), stabilizing (Preziosi & Fairbairn, 2000; Schluter & Smith, 1986), disruptive (Gross, 1985), and fluctuating (Bonnet & Postma, 2018; Lisle Gibbs & Grant, 1987; Seamons, Bentzen, & Quinn, 2007) selection on body size or related traits. Salmonid fishes provide excellent model systems in this regard since many of their populations are intensively studied, body size metrics are often routinely measured, and fitness components can be measured directly (Carlson & Quinn, 2007; Carlson, Rich, & Quinn, 2009; Kendall, Hard, & Quinn, 2009; Morrissey & Ferguson, 2011; Quinn, Hendry, & Buck, 2001) or estimated indirectly using molecular pedigrees (Aykanat et al., 2014; Seamons et al., 2007; Naish, Seamons, Dauer, Hauser, & Quinn", 2013; Reed et al., 2018; this study).

Large size is generally expected to be advantageous to both female and male anadromous salmonids, but for different reasons. Larger females can produce more and larger eggs (Bacon, MacLean, Malcolm,& Gurney, 2012; Beacham & Murray, 1993; de Eyto et al., 2015), can dig deeper nests so that their eggs are less susceptible to scouring in high river flows (Steen & Quinn, 1999), and compete better for limited nest sites (Holtby & Healey, 1986). Selection pressure in males, on the other hand, may be more influenced by sexual selection for access to mates, with larger males better able to court and defend females (Fleming, 1996; Fleming & Gross, 1994). However, small “sneaker” males persist as an evolutionarily stable strategy in some systems, as they are able to “steal” fertilizations from larger, more socially dominant males (Fleming & Einum, 2011). Previous studies on Pacific salmonids have found sex differences in the form and magnitude of selection on adult body size (Carlson & Quinn, 2007; Fleming & Gross, 1994; Seamons et al., 2007).

Using measures of total adult‐to‐adult fitness (individual lifetime reproductive success, *LRS*) inferred from a molecular pedigree for nine cohorts of spawning adult Atlantic salmon, our aims were to determine (a) whether adult body size was, on average, under linear and/or nonlinear selection across the considered time period, and (b) its evolutionary potential. Having shown the trait to be both heritable and under directional selection in females, our subsequent goals were to (c) test for a microevolutionary trend in female body size over time and (d) explore whether the observed evolutionary response was concordant with expected responses to selection predicted using two theoretical approaches: the Breeder's Equation (BE; Lush, 1937) and the Robertson–Price Identity (Robertson, 1966, 1968; Price, 1970; also known as the secondary theorem of selection, hereafter STS). The BE can give biased predictions if there are variables missing from the analysis that covary with both the focal trait and the fitness (Hadfield, 2008; Morrissey et al., 2010). The STS provides an estimate of the expected evolutionary change in mean trait value per generation, given by the additive genetic covariance between trait and relative fitness, which is unbiased by missing traits or environmental variables (Hadfield, 2008; Morrissey et al., 2010). A comparison of BE versus STS predictions can, therefore, be a useful indirect test of the presence of such missing traits or environments, particularly if observed evolutionary responses are more concordant with STS than with BE predictions. Our final aim was to (e) use the more direct method of Rausher (1992; see also Hadfield, 2008, Morrissey et al., 2010; Morrissey et al., 2012, and Stinchcombe et al., 2014) to quantify the difference between genetic and nongenetic regressions of fitness on trait, which if present would bias evolutionary predictions based on the univariate BE.

## 2 METHODS

### 2.1 Study system

The Burrishoole catchment in the West of Ireland (Figure S1) drains an area of approximately 100 km^2^ of varying topography and land use (de Eyto et al., 2016) and consists of three major lakes: brackish Lough Furnace, connected to the sea by the Burrishoole River, and the larger, freshwater Lough Feeagh and Bunaveela, with Atlantic salmon spawning in a series of afferent rivers. A total trapping system operates on the catchment, where all upstream migrating prespawning adults, all downstream migrating postspawning adults (kelts), and all downstream migrating juveniles (smolts) are enumerated. Traps are located on two short rivers that connect Lough Feeagh to Lough Furnace (Figure S1). A hatchery has operated on the catchment since the early 1960s as part of an experimental ocean ranching program (McGinnity et al., 2009). The hatchery rears a core “ranched” salmon stock, that originated from wild Burrishoole fish, up to the smolt stage, at which point they are usually released into Lough Furnace and allowed to migrate to sea naturally (although historic releases into Lough Feeagh and directly into the estuary have also occurred). Starting in the 1960s, returning hatchery fish (identified by an adipose fin clip) were externally tagged and allowed to migrate upstream. Subsequent downstream homing behavior allowed a proportion of these to be removed, with a subsample of these fish used as broodstock for the following generation of captive‐reared salmon. In recent years, the management goal has been to reduce the proportion of hatchery fish to less than 5% of the spawning stock that is allowed to ascend the traps to spawn. Therefore, varying numbers of hatchery fish have been released above the traps over the years, some of which spawn in the wild (Aykanat et al., 2014; McGinnity et al., 2009; Thompson, Poole, Matthews, & Ferguson", 1998). Thus, there has been some gene flow from the hatchery to the wild population. This study focusses exclusively on wild‐spawning fish, that is, fish who were born in the wild or in the hatchery, but who themselves spawned in the wild; the evolutionary dynamics within the hatchery are not examined.

### 2.2 Pedigree construction

Microsatellite genotype data were used to construct a molecular pedigree of all returning fish, using the Cervus software 3.0.7 (Kalinowski, Taper, & Marshall, 2007). Full details on fish sampling, DNA extraction, genotyping, and pedigree construction protocols are provided in Aykanat et al. (2014) and in Appendix 1. The sex of returning fish was determined based on phenotypic characteristics and confirmed genetically with a sex marker (see Aykanat et al., 2014 and Appendix 1 for details).

The term “cohort” is hereafter used to refer to the year a fish returned from the sea on its spawning migration; note that fish may return over a range of months, from June to September, and most spawn in December, but some spawning may also occur the following year in January. While a pedigree was constructed from all available data (Table S1), due to breaks and changes in the sampling regime since the 1960s, not all years could be included in the analyses described herein. We report identity analysis results and the false discovery rate for the entire pedigree (Figure S2, Table S2). After data cleaning (see code at https://doi.org/10.20393/1b6fed63-4d4b-40f5-9473-32e8210e605a, O'Sullivan et al., 2019), the pedigree used in this study comprised of wild‐spawning fish for the following cohorts: 1977, 1978, 1979, 1980, 1981, 1982, 1984, 1985, and 1989 (Figure S3). On average, 90% of fish in this system follow a four‐year lifecycle (Piggins & Mills, 1985): Individuals will spend two years in freshwater, migrate to sea for one winter, and then return to the catchment in the following year to spawn. There is some generational overlap (see Figure S4 for a diagrammatic explanation of the typical four‐year lifecycle). For example, fish spawning in 1989 represent the offspring of fish that would have spawned mostly in 1985, but with a small fraction coming from 1983, 1984, and 1986. The offspring of fish spawning in 1989 would themselves return and be sampled as adults predominantly in 1993. A gap in sampling in 1991, 1992, and post‐1993 precluded us from being able to determine whether we missed any offspring spawned by the 1989 cohort that did not recruit in 1993, and so *LRS* may be underestimated for fish that spawned in 1989. Selection analyses were re‐run excluding data from the 1989 cohort, and the results were qualitatively unchanged; hence, this potential source of bias was deemed unproblematic.

In this study system, up until 2011, upstream migrating adults were enumerated but not sampled for DNA or measured for phenotypes. Instead, they were sampled as kelts in the traps on their postspawning downstream migration back to sea. This sampling regime aimed to avoid stressing the fish on their upstream spawning migration. However, periodic sampling of upstream migrating fish did occur in some years (e.g., 1977, 1978). Some mortality occurs in freshwater either prior to, during, or postspawning, with mortality much higher in males, leading to a female bias in our sample (Aykanat et al., 2014). On average across the whole study, the number of fish measured for fork length (hereafter referred to simply as body size) represented approximately 50% of the total numbers of upstream migrating prespawners. While adults lose mass between entering freshwater and leaving again after spawning, adult female skeletal size is not expected to change; thus, body size of female kelts can safely be assumed to reflect body size at spawning. As such, all sampled females from the relevant cohorts were used in the estimation of the female size selection gradient. Since male skeletal length is known to increase between freshwater entry and spawning (due to the development of a secondary sexual characteristic of the jaw known as the “kype”), selection analysis on male length was limited to only those males that were sampled as kelts. We also assume that kelts represent a random subset of original spawners with respect to body size and *LRS*, but we lack the data to formally test this and explore in the Discussion the possible implications of violations to this assumption.

The *LRS* of each fish was measured by counting the number of offspring assigned genetically to that individual who themselves returned as adults in future years and in turn were sampled. We acknowledge that this *LRS* measure is potentially an underestimate of lifetime fitness given that some returning adults (in particular males) die prior to being sampled as kelts, while a small fraction of adult offspring may “stray:” (i.e., return to rivers other than their natal river), but this need not lead to biased microevolutionary inferences (see Discussion). The final dataset consisted of 1,185 records of female *LRS* and body size, and 302 records of male *LRS* and body size (with no repeat measures in either sex; note that while Atlantic salmon are capable of iteroparity, this is rare in our study system) measured across nine return cohorts (Table S3).

### 2.3 Phenotypic selection

Body size for each fish was mean and variance standardized (hereafter denoted as *Size’*) by subtracting the overall grand mean body size across the nine cohorts from each individual body size measure and dividing by the overall standard deviation. This yields a standardized size measure known as a “*z*‐score.” Using *z*‐scores allows for the estimation of standardized selection coefficients which are directly comparable across studies (Lande & Arnold, 1983). This was done separately for males and females, which varied in their means and standard deviations, and selection analyses were performed separately since selective regimes are known to differ between the sexes in salmonids (Fleming, 1996; Seamons et al., 2007). Overall patterns of linear and nonlinear phenotypic selection across the whole study period were estimated for each sex separately using generalized linear mixed effects models (GLMMs) implemented in the “MCMCglmm” R package (Hadfield, 2010; R Core Team, 2017), in which *LRS* was the response variable and the explanatory variables included linear and quadratic effects of *Size’*. Models were fit using a Poisson error structure, and a log‐link function as MCMCglmm's Poisson error structure automatically accounts for overdispersion in the data. We derived linear and quadratic selection coefficients using the method of Morrissey and Goudie (2016). See https://www.biorxiv.org/content/10.1101/040618v1). Briefly, this method estimates linear and quadratic selection coefficients from GLMMs that are equivalent to those estimated from standard Lande–Arnold regressions. We focus on a single trait expressed as *z*‐scores and so regression coefficients in the selection analyses correspond to both standardized selection differentials and univariate standardized selection gradients (Postma, 2006). Hereafter, these are referred to as selection gradients, but noting that they do not necessarily reflect true direct selection on body size, as correlated traits affecting fitness could be missing from the analyses (Lande & Arnold, 1983). Selection analyses used MCMCglmm's default priors, equivalent to a Gaussian distribution for the fixed effects, and an inverse‐gamma distribution for the variances (see code at https://doi.org/10.20393/1b6fed63-4d4b-40f5-9473-32e8210e605a).

### 2.4 Animal models to estimate quantitative genetic parameters

Initial exploration of male quantitative genetic parameters was impeded by small sample sizes and large associated errors. As such, all further quantitative genetic analyses were conducted solely on females. First, we ran a univariate animal model with *Size’* as the response variable, an intercept as the only fixed effect, and random effects that included an additive genetic effect (with the variance in these corresponding to the additive genetic variance, *V*_A_), a maternal effect (*V*_dam_), a cohort effect (*V*_cohort_), and a residual effect (*V*_resid_). Narrow‐sense heritability (*h* ^2^) was then calculated by dividing *V*_A_ by the sum of all variance components (*V*_A_ + *V*_dam _+ *V*_cohort_ + *V*_resid_). No fixed effects were included in the analysis as no additional individual‐specific information was available on environmental variables or traits that might influence body size, such as the date a fish was sampled. Female Atlantic salmon stop feeding once they return to freshwater and are therefore not expected to either gain or lose skeletal size during the adult freshwater phase. While there is variation in date of ocean exit, that is, “run timing,” which may be associated with variation in body size (Quinn, McGinnity, & Cross, 2006), we had no individual‐level information on this. Sea age—the number of winters spent at sea prior to freshwater return—is also correlated with body size at return and is itself heritable in Atlantic salmon (Barson et al., 2015; Reed et al., 2018). Given that over 90% of fish in this population return after a single winter at sea (known as “grilse”) and that the inclusion of heritable traits as fixed effects can affect estimates of *V*_A_ for the focal trait, sea age was not included as a fixed effect in the animal models. The animal model for *Size’* was initialized with a burn‐in period of 500,000 iterations and then run for a further 2,000,000 iterations, with a thinning interval of 1,000, giving a final MCMC sample size of 2,000.

For a trait to respond to selection, there must be additive genetic variance in the trait, as well as a covariance between fitness and the trait (Fisher, 1930). As such, we estimated *V*_A_ and *h* ^2^ for *LRS* using an animal model with the same fixed and random effects structure as that used for *Size’*. The “QGglmm” R package was used to integrate over the posterior distributions of the random effects for the animal model of *LRS*, in order to convert the estimated variance components from the latent scale to the observed scale of the data (Bonnet & Postma, 2018; de Villemereuil, Schielzeth, Nakagawa, & Morrissey", 2016). The animal model for *LRS* was initialized with a burn‐in period of 1,000,000 iterations and then run for a further 14,000,000 iterations, with a thinning interval of 10,000, giving a final MCMC sample size of 1,400. Univariate animal models used noninformative, parameter‐expanded priors (see code at https://doi.org/10.20393/1b6fed63-4d4b-40f5-9473-32e8210e605a).

### 2.5 Testing for observed microevolutionary change

Conceptually, a microevolutionary change occurs within a population when the mean breeding value—a measure of the “genetic merit” (additive genetic effects) of individuals for the trait of interest—changes over time. Predicted breeding values for *Size’* for each individual were extracted from the female univariate animal model, and the observed temporal change in mean breeding values (slope of mean annual breeding value vs. cohort as a continuous variable) across the study period was calculated using a variant of the method described in Hadfield, Wilson, Garant, Sheldon, and Kruuk (2010). We fitted *Cohort* as a random effect as per Postma (2006), rather than as a fixed effect as per the Hadfield method. This gave a posterior distribution of temporal slopes of estimated mean breeding values (EBVs) which corresponds to a distribution of estimates for the linear rate of evolutionary change. We a priori expected a positive microevolutionary trend, given that positive directional selection was found for females (see Results), and statistical support for this was assessed by calculating the fraction of the posterior distribution of temporal slopes that was greater than zero. The probability that the observed change in EBVs was different from a scenario of genetic drift was then calculated by simulating random breeding values for *Size’* down the pedigree using the *rbv*() function in MCMCglmm (Hadfield, 2010) for each of the 2,000 posterior samples of the univariate animal model for *Size’* based on the estimated *V*_A_. Linear regressions were fitted to the cohort mean of these random breeding values to obtain the temporal slopes due to drift for each posterior sample. The fraction of the posterior distribution of observed temporal slopes that was greater than these “drift slopes” was then calculated. This provides an estimate of the probability that the observed microevolutionary trend was greater than expected due to genetic drift alone. Since *Size’* was a mean and variance standardized quantity and was regressed on years, the units for evolutionary change here were phenotypic standard deviations per year (PSD). The estimated rate of microevolution on an annual basis was converted to a per‐generation rate, by multiplying the annual rate by four (the average generation time of fish in our study system). This is then equivalent to a change measured in “Haldanes” (PSD per generation).

### 2.6 Comparing observed microevolutionary change against predictions from the BE and STS

The expected per‐generation rate of adaptive evolutionary change for this population was first calculated based on the univariate BE (Equation 1):

(1) $$R_{\text{BE}}=V_{A}\beta$$

where *R*_BE_ = the response to selection, that is, the predicted genetic change in the mean trait value from one generation to the next based on the BE, and *β* is the univariate selection gradient, which in our case corresponds to the linear coefficient for body size on the latent scale in the phenotypic selection analysis. To obtain a full posterior distribution of *R*_BE_ that accounts for all uncertainties in the estimation procedures, we multiplied realizations of *V*_A_ and *β*_Size_ from their respective posteriors to obtain samples from the posterior of *R*_BE_ and then calculated the posterior mode and 95% credible intervals for *R*_BE_ from this. This allowed us to determine whether the per‐generation rate of *observed* microevolution, as calculated in the previous step, matched the *predicted* response derived from the BE.

The STS states that the additive genetic covariance (cov_A_) between a trait (*z*) and relative fitness (*w*) is a direct measure of the expected per‐generation evolutionary change in that trait, unbiased by unmeasured covariates (Price, 1970; Robertson, 1966; Stinchcombe et al., 2014). We call this an evolutionary “response” for linguistic consistency but recognize that the STS is agnostic regarding the drivers of evolutionary change, which could include drift or selection on a genetically correlated trait, in addition to direct selection on the trait itself.

(2) $$R_{\text{STS}}=cov_{A}\left(w,z\right)$$

We defined a bivariate animal model with *Size’* and *LRS* as response variables to estimate the additive genetic covariance between them, which in this case corresponds to cov_A _(*w*, *z*) due to the log‐link function on *LRS* (Morrissey & Goudie, 2016). The bivariate animal model consisted of an intercept with random effects for additive genetic, dam, cohort, and residual effects specified within an unstructured variance–covariance matrix, using noninformative, parameter‐expanded priors. As before, this gives a full posterior distribution for *R*_STS_, for which we report the posterior mode and 95% credible intervals. For all models, Markov chains were thinned so as to keep autocorrelation between successive draws below 10%. Alternative priors were specified for all models (selection and animal), with none proving sensitive. See code at https://doi.org/10.20393/1b6fed63-4d4b-40f5-9473-32e8210e605a for details of priors.

### 2.7 Quantifying bias in phenotypic selection gradients

Work by Rausher (1992), Hadfield, (2008), Morrissey et al., (2012), and Stinchcombe et al., (2014) has shown that the difference between the “environmental selection gradient,” *β*_E_ (which corresponds conceptually to the regression slope of environmental deviations for fitness on environmental deviations for trait) and the “genetic selection gradient,” *β*_G_ (which corresponds conceptually to the regression slope of breeding values for fitness on breeding values for trait) provides a metric of so‐called “environmental bias” to phenotypic selection. For example, a purely environmental variable such as nutritional status might influence both the focal trait and fitness, generating phenotypic covariance between them even if the trait does not necessarily causally influence fitness (Price, Kirkpatrick, & Arnold, 1988). The phenotypic selection gradient would be biased, in the sense that there is no selection on underlying breeding values in this hypothetical example, nor would any response to selection be expected even if the trait were heritable (Rausher, 1992). While this is typically referred to as “environmental bias,” phenotypic selection estimates may be biased whenever there are unmeasured factors of any sort, be they genetic or environmental, which correlate with both focal trait and fitness (Hadfield, 2008; Morrissey et al., 2012). The difference (in slopes) between nongenetic and genetic regressions of fitness on trait represents our “bias statistic” (hereafter, referred to as “∆*β*”), and because we used a Bayesian approach, we could obtain a posterior probability that this bias statistic was greater than zero, which would imply stronger positive “selection” at the nongenetic, compared to the genetic, level. This in turn can be interpreted as the probability that predictions from the univariate BE are biased by missing traits or environments.

We had only a single focal trait and so *β*_G_ and *β*_E _could be calculated from the bivariate animal model of trait (*Size’*) and fitness (*LRS*) used to calculate *R*_STS_. For *β*_G_, this involved dividing *cov*_A_ (*w*, *z*), equivalent to a genetic selection differential, by *V*_A_, to give a univariate selection gradient. To calculate *β*_E_, we summed all the environmental covariance terms in the bivariate animal model and divided by the sum of the corresponding variance components for *Size*’. The bias statistic, ∆*β*, was then calculated as *β*_E _− *β*_G_, using full posterior distributions for each (Morrissey et al., 2012). When the 95% credible intervals of the resulting posterior distribution of ∆*β* do not include zero, there is sufficient evidence to state that there is bias in the phenotypic selection measure. If the credible intervals include zero, there is insufficient evidence to suggest bias, but equally one cannot conclude unequivocally that there is no bias in situations where statistical power may be low (Reed et al., 2016; this study).

The results for all parameter estimates from our Bayesian models are expressed as posterior modes and 95% highest posterior density (HPD) intervals. Variance components by definition cannot be negative but were deemed statistically not significant when the lower HPD interval overlapped zero.

## 3 RESULTS

### 3.1 Phenotypic selection

**Figure 1:**
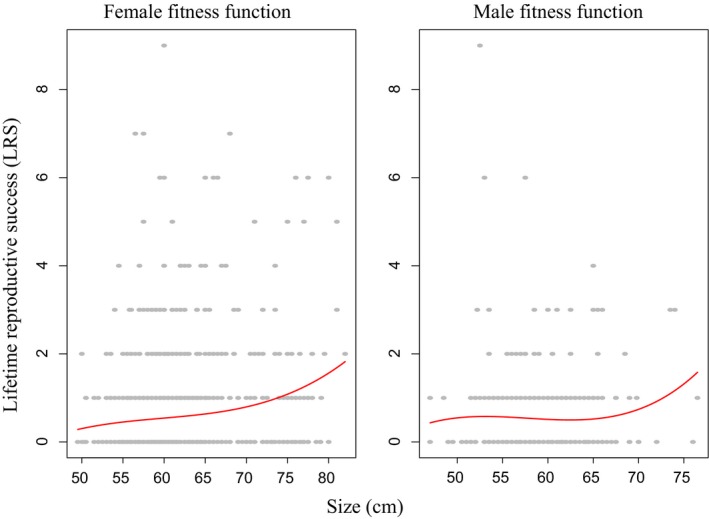
Phenotypic selection patterns (red curves) on body size measured in centimeters (cm) for female and male Atlantic salmon. Selection gradients were approximated for illustration purposes using univariate cubic splines c.f. Schluter (1988) and Wilson, Pilkington, et al. (2005)

Phenotypic selection was positive in females, with credible intervals that did not overlap zero (*β*_Size_ = 0.23, 95% HPD: 0.08, 0.34), implying directional selection for larger body size (Figure 1, Table 1). The quadratic selection gradient was close to zero with credible intervals including both negative and positive values (Table 1), implying weak or no nonlinear selection in females. For males, the linear selection gradient was less than a third that of females, with credible intervals including a broad range of negative and positive values (Figure 1, Table 1), suggesting either a lack of consistent directional selection in males or insufficient statistical power to detect a real relationship (given that sample size for males was only 302, compared to 1,185 for females). Similar to females, the quadratic selection gradient was close to zero in males with credible intervals including both negative and positive values (Table 1).

**Table 1:** Linear and quadratic standardized (univariate) selection gradients for female and male Atlantic salmon

	Female		Male	
	Posterior mode	95% HPD	Posterior mode	95% HPD
Linear selection	0.23	0.08 to 0.34	0.07	−0.18 to 0.26
Quadratic selection	0.1	−0.005 to 0.24	0.04	−0.12 to 0.56

### 3.2 Univariate animal models

The animal model for *Size’* revealed significant additive genetic variation in female *Size’*, as well as significant cohort and residual effects, with the maternal effect being very close to zero (Table 2). *LRS* showed significant cohort and residual effects, with the additive genetic and maternal effects being very close to zero. Heritability (*h* ^2^) of female *Size’* was estimated at 0.23 (95% HPD: 0.06, 0.41). After transformation from the latent to the data scale, *h* ^2^ for *LRS* was estimated as 0.0005 (95% HPD: <0.0001, 0.11).

**Table 2:** Posterior modes and 95% HPD intervals for additive genetic variance (*V*_A_), cohort (*Cohort*), maternal (*Dam*), and residual (*Residual*) variance component estimates from univariate animal models for *Size'* and *LRS* in female Atlantic salmon

Parameters	Size'					LRS				
	*V*_A_	Cohort	Dam	Residual	*h* ^2^	*V*_A_	Cohort	Dam	Residual	*h* ^2^_Data scale_
Posterior mode	0.26	0.08	0.0003	0.68	0.23	0.003	0.33	0.001	0.93	0.0005
95% HPD	0.05–0.42	0.03–0.35	<0.0001–0.1	0.51–0.89	0.06–0.41	<0.001–0.57	0.1–1.21	<0.001–0.18	0.4–1.25	<0.0001–0.11

Note

Heritability (*h* ^2^) estimates were calculated as the quotient between *V*_A_ and the sum of *V*_A_, *Cohort*, *Dam*, and *Residual*. For *LRS*, all parameter estimates are given on the latent scale, with the exception of *h* ^2^, which is on the data scale after integration over the variance components (see Methods).

### 3.3 Comparing observed versus predicted evolution

**Figure 2:**
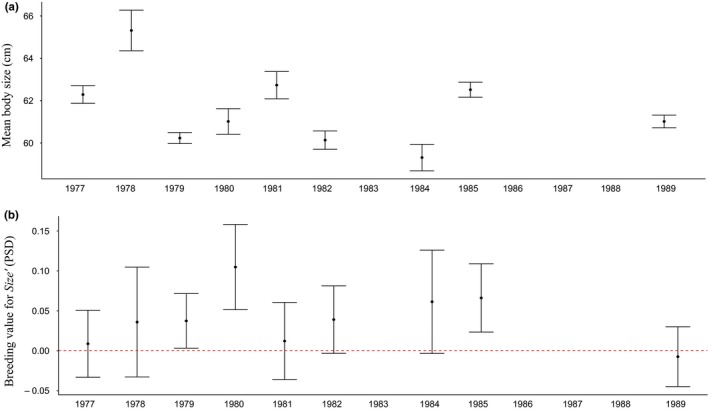
(a) Observed phenotypic trend in mean body size for female Atlantic salmon over the period 1977–1989. The upper and lower bounds of the whisker plots represent standard errors; (b) observed evolutionary trend in cohort mean breeding values for Size' (measured in phenotypic standard deviations, PSD, with the standardization done using the global mean and standard deviation) in female Atlantic salmon over the period 1977–1989. The upper and lower bounds of the whisker plots represent standard errors

**Figure 3:**
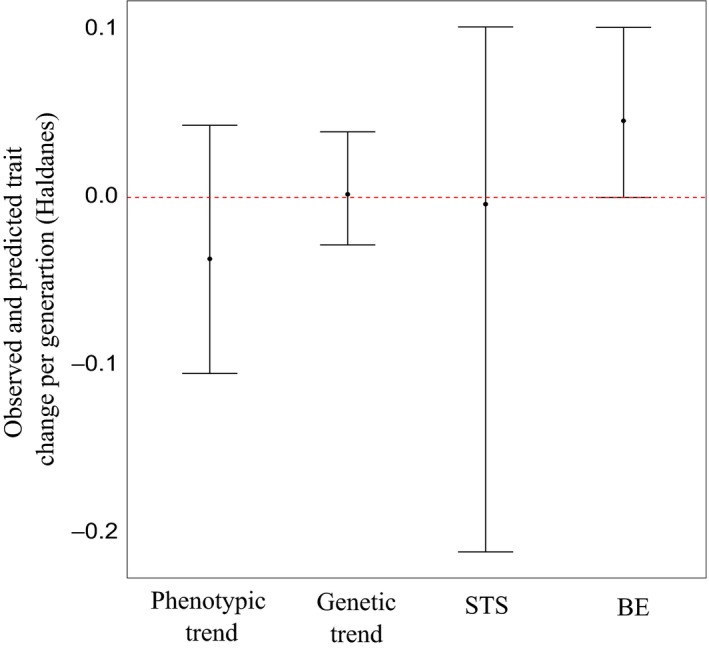
Comparison of observed and predicted trends in Size' in female Atlantic salmon, with predicted evolutionary trends in breeding values based on the univariate Breeder's Equation (BE) and the Secondary Theorem of Selection (STS). The observed evolutionary change (Genetic trend) was determined by extracting estimated breeding values from the univariate animal model for Size' and testing for a temporal trend

There was no overall temporal trend in annual mean phenotype across the 1977 to 1989 study period (−0.18 cm/year; 95% HPD: −0.55, 0.24; Figure 2a). Likewise, there was no genetic trend in EBVs for female body size (0.0005 PSD per year, 95% HPD −0.007, 0.01; Figure 2b), with the posterior probability of this trend being greater than zero being only 59%. The probability of the temporal trend being more positive than expected under a scenario of genetic drift was 57%. Re‐expressed in phenotypic standard deviations per generation (Haldanes) rather than per year, this corresponded to an observed per‐generation evolutionary change of 0.002 Haldanes (95% HPD: −0.03, 0.04; Figure 3). By comparison, the BE predicted a per‐generation rate of evolutionary change in female body size of 0.05 Haldanes (95% HPD: <0.001, 0.10; Figure 3), implying that female salmon were predicted to increase in size across the time period. The STS, on the other hand, predicted a rate of evolutionary change in female body size of −0.004 Haldanes, with credible intervals broadly overlapping zero (95% HPD: −0.21, 0.10; Figure 3); that is, it predicted a lack of any consistent response to selection, which was concordant with the observed lack of temporal trend in estimated breeding values or mean phenotype.

### 3.4 Quantifying bias in selection gradients

**Figure 4:**
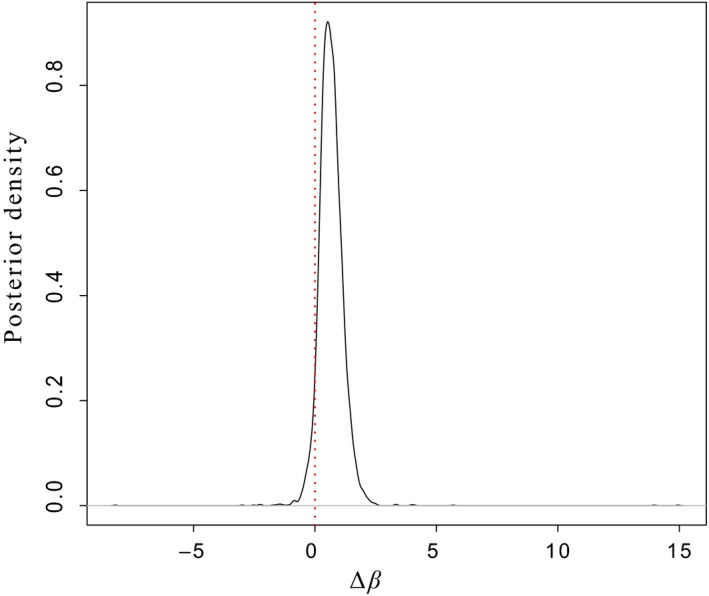
The posterior distribution of the bias statistic, Δ*β*, between environmental and genetic selection gradients. 94.2% of the distribution lies above zero (right of the dashed vertical line), strongly suggesting bias in the female phenotypic selection gradient due to unmeasured trait(s) or environmental factors

The posterior mode estimate for Δ*β* was 0.43, indicating that missing traits or environmental variables contribute to a more positive association between trait and fitness than can be attributed to the effect of the trait alone on fitness. Credible intervals overlapped zero (95% HPD: −0.21, 1.7: Figure 4) with 94.2% of the slope estimates greater than zero.

## 4 DISCUSSION

### 4.1 Selection analyses

Based on information derived from nine cohorts spanning three generations of our molecular pedigree, we found evidence for positive directional selection on female body size in Atlantic salmon. There was no evidence for directional selection on male body size, nor for nonlinear selection in either sex. Our finding of positive directional selection on female body size was consistent with predictions (Fleming, 1996) that larger female salmon should experience greater reproductive success for myriad potential reasons (e.g., produce more eggs, produce larger eggs that give an early size advantage in offspring, more aggressive, secure better territories). While the ecological drivers of this positive selection in females remain unclear, size‐mediated competition among the adults for suitable spawning sites and among offspring for territories is likely to be involved. Seamons et al. (2007) also documented positive linear selection on body size (fork length) in anadromous female steelhead trout (*Oncorhynchus mykiss*) using pedigree‐derived *LRS* as the fitness measure. Adult‐to‐adult *LRS* for females can be decomposed into three components: mating success, fecundity, and offspring viability (egg‐to‐adult survival). Maternal body size could in theory affect all three components, but effects on offspring viability would be indirect and mediated via factors such as egg size and physical qualities of the nest site (Fleming, 1996). As such, maternal effects on offspring survival are likely limited to early stages (egg to fry), attenuating thereafter at the juvenile, smolt, and marine phases (Reed et al., 2015). Stochastic environmental effects probably dominate variation in overall egg‐to‐adult survival, and hence, it is unsurprising that maternal body size explains so little of the variation in *LRS*. The theoretical and practical implications of assigning offspring viability as a component of maternal fitness are discussed further below.

For males, direct effects of body size on *LRS* likely act solely via mating success, although indirect effects may arise if there is positive assortative mating, where large males mated to large females sire more or better quality offspring (Fleming, 1996). As such, the overall lack of evidence for selection on male body size in our study is intriguing. Theory suggests that male Atlantic salmon should experience disruptive selection: Large ocean‐going (anadromous) males and early‐maturing males (sneakers) that spawn before going to sea are predicted to achieve higher fitness than intermediate‐sized males, which cannot compete as successfully against larger anadromous males for access to females, nor adopt as effectively the sneaking tactic of smaller mature males (Hutchings & Myers, 1994; Taborsky, 2008). While these may be different “traits” in the sense that different genes/developmental pathways might affect size‐at‐first‐maturity of anadromous males versus early‐maturing males, recent work shows that the same QTL may influence both sea age at maturity (Ayllon et al., 2015; Barson et al., 2015) and early male maturation (Lepais, Manicki, Glise, Buoro, & Bardonnet, 2017). Our study was limited to anadromous males only, and while some may have spawned previously as sneaker males, these fish would not have been sampled at that stage. As up to 30% of paternities in Burrishoole may be attributed to sneaker males, approaching 60% in years of proportionally high hatchery spawning (Thompson et al., 1998), disruptive selection could well occur across the full range of male body sizes.

Another explanation for the lack of selection in males could simply be that there is little variation in male size in this population, and hence a reduced scope for selection. For example, Atlantic salmon in the River Teno/Tana in Finland/Norway exhibit a much larger range of male body sizes, and selection for larger males is known to occur there (Mobley et al., 2019; see also Fleming, 1998). Anthropogenic changes over the past several decades, particularly in the marine environment, have reduced the prevalence of larger, older salmon in some populations across their range (Chaput, 2012; Quinn et al., 2006; Reed et al., 2017), including the Burrishoole (Nixon, 1999), which may in turn limit the opportunity for ongoing selection.

Among anadromous males, we still expected to find positive directional selection given that larger males may have an advantage in intrasexual competition in Atlantic salmon (Hutchings & Myers, 1987) and other salmonids (Fleming & Gross, 1994; Quinn, Hendry, et al., 2001). Our sample size of 302 males may have been too small to detect subtle directional selection. Or it may be there are costs of larger body size (e.g., increased aggression from other males during establishment of dominance hierarchies, predation costs) that counteract any sexually selected benefits. A further hypothesis is that females may choose males on the basis of traits which are uncorrelated with overall body size. Selection pressures are also likely to be context‐specific; for example, Seamons et al. (2007) found that larger male steelhead trout had higher *LRS* on average than smaller males, but the strength of selection varied among years for unknown reasons. In contrast, Carlson and Quinn (2007) documented selection against larger male sockeye salmon (*Oncorhynchus nerka*) in an Alaskan study population, with the largest males (and females) being more susceptible to stranding at the mouth of the spawning river connected to a lake, particularly in years where lake levels were low. Larger fish in that system are also more susceptible to brown bear (*Ursus arctos*) predation (Quinn, Wetzel, Bishop, Overberg, & Rogers", 2001).

If straying rates in our system are correlated with both body size and fitness, then our estimates of selection on body size could be biased in a global sense; that is, different relationships between size and fitness might have been found in either sex if the body size and *LRS* of strayers into non‐natal rivers could be measured and included in the analysis. Conceivably, strayers may be a nonrandom subset of the local population in this regard; however, we have no reliable data on this. Selection estimates would only be biased if the relationship between trait and fitness was not the same in strayers versus nonstrayers. Similar issues arise in nestbox population studies of passerine birds, where study areas typically represent only a small local sample of a much larger, widespread population. In such situations, selection estimates are best interpreted at a local scale; that is, they represent the relationship between phenotype and *local* recruitment. Local selection pressures and their consequences for discrete, locally adapted salmonid populations such as our study system (O'Toole et al., 2015) are arguably of more interest than estimates of global selection, unless one is interested specifically in meta‐population dynamics.

A second source of methodological bias could arise from the fact that, for males, we were limited to sampling postspawning kelts, rather than prespawning adults on their upriver migration. For example, if larger males were more likely to die on the spawning grounds but also experienced higher *LRS* than smaller surviving males, then our male selection estimates would be biased downwards (an example of the “invisible fraction” problem sensu Grafen, 1988; see also Hadfield, 2008). We are unable to explore this potential source of bias as we almost always only sampled males as kelts that, by definition, survived the spawning period. This problem may be male‐specific, as spawning survival rates for females are much higher (55%–80%; Anon.) —as evidenced by our much higher sample sizes for females relative to males (Table S3).

### 4.2 Quantitative genetic parameters and observed versus expected evolutionary dynamics

Although heritability estimates are by their nature population‐ and environment‐specific, our estimate of heritability for female body size was similar to previously published estimates for this trait in adult Atlantic salmon (*h* ^2 ^= 0.32 in Saura et al., 2010; *h* ^2 ^= 0.27 in Reed et al., 2018) and fell within the range of previous estimates for heritability of size during juvenile stages within our system (Reed et al., 2015). More generally, our estimate of body size heritability was congruent with the median estimate of 0.21 reported by Carlson and Seamons (2008) for morphological traits across 11 salmonid species. Dam effects on body size were effectively nonexistent: When expressed as a percentage of the total variation, *V*_dam_ only explained ~0.022% (Table 2). This was unsurprising, in that maternal effects on offspring traits are expected to attenuate with offspring age in salmonids (Heath, Fox, & Heath, 1999; Reed et al., 2015), such that by the time the offspring is an adult, there is almost no discernible maternal effect remaining. Among the remaining phenotypic variation not attributable to additive genetic or maternal effects, cohort effects accounted for ~7.2% and residual effects for ~67% (Table 2). This implies that environmentally driven variation in growth among individuals within years is greater than between‐year variation, which is largely driven by marine growth.

Our univariate animal model for *LRS* revealed a very low *h* ^2^ for this fitness trait, with a modal estimate that was close to zero. The low *h* ^2^ for *LRS* reflected very low *V*_A_ for *LRS*, with the posterior distribution of *V*_A_ similarly abutting zero and having a long right tail. There may be very little segregating genetic variation in fitness in this population, which is what one would expect theoretically at equilibrium (Fisher, 1930), unless balancing selection mechanisms or a high mutational target maintain genetic variance in fitness (Houle, 1998). Low *h* ^2^ for fitness does not necessarily imply low *V*_A_, however, as various stochastic environmental and demographic processes can lead to very high environmental sources of fitness variance which can dominate in the calculation of *h* ^2^ (Kruuk et al., 2000). Our animal model for *LRS* contained a log‐link function, making *V*_A_ of fitness interpretable as the genetic variance of relative fitness. While our estimate of this was modest (0.003), the 95% HPD contained nontrivial values which may represent the true parameter value. If *V*_A_ in relative fitness is indeed rather low in our salmon population, this may provide a partial explanation for our observed evolutionary stasis: There can be no genetic covariance between body size and fitness, that is, microevolution, if there is no genetic variance in fitness (Orr, 2009).

Indeed, the observed lack of microevolution was consistent with the predicted rate according to the STS being effectively zero. As explained in Morrissey et al. (2010) and Morrissey et al. (2012), the STS provides a more robust, less assumption‐laden guide to expected microevolution in natural populations than the BE, although it is not itself completely without problems: Various ecological complications such as spatio‐temporal variation in the expression of genetic variation, nonrandom migration, and nonconstant demographic structure may render STS predictions inaccurate. Given the partial agreement between the STS prediction and our observed evolutionary stasis, we tentatively conclude that our study is not hampered by such complications.

The BE in both its univariate and multivariate forms assumes that all relevant traits have been included in the analysis (Lande & Arnold, 1983). A necessary condition for the univariate BE to always produce accurate evolutionary predictions is that the focal trait must be the *sole* cause of covariation between phenotype and fitness. In the case of the multivariate BE, the key assumption is the presence of what Morrissey et al. (2010) call “joint‐sole’’ causation; that is, the traits included in the analysis are collectively the only traits determining phenotype‐fitness covariance. In practice, these assumptions can be rather restrictive in natural populations, where entire suites of traits may be under selection and intercorrelated to varying degrees. Failure to include any of these traits in a multivariate BE analysis may render the results biased. Adult body size in salmon is likely to be correlated with other traits such as return timing, sea age, or aspects of intrinsic metabolic rate, which may each experience different, potentially antagonistic, direct selection pressures. This is the reason, we believe, why our univariate BE prediction suggested a positive directional response to selection, whereas the STS prediction was equivocal, with the potential for either a positive or a negative evolutionary change in body size. However, we are cautious not to overinterpret this comparison between STS and BE predictions, because both were associated with rather large 95% credible intervals, likely due to our relatively low sample sizes and shallow pedigree. Thus, while the posterior mode of the STS prediction was close to zero (−0.004 Haldanes), the upper credible interval was higher (0.10 Haldanes) than the posterior mode for the BE prediction (0.05 Haldanes). It remains possible, therefore, that both approaches actually predict positive directional evolution in this system, but there is insufficient statistical power to conclude the STS prediction is different from zero. The fact that our comparison of selection at the genetic versus environmental levels provided reasonably strong support for a bias (i.e., missing traits or environments), and that there was also no evidence for any observed microevolutionary trend, points toward a scenario of true evolutionary stasis that is correctly predicted by the STS but not the BE. But the statistical power to detect relatively subtle evolutionary trends was likely low, so again we cannot outright reject a scenario of true directional evolution that would be correctly predicted in sign (but not necessarily magnitude) by both the STS and BE approaches if the sample sizes had been higher and/or the pedigree was deeper. These caveats must be born in mind in interpreting our results, and if any of our estimates are to be used in meta‐analyses, we recommend that they are appropriately weighted by their large uncertainty.

### 4.3 Quantifying bias in selection gradients

Our estimation of the probability of bias in the female phenotypic selection gradient (i.e., the probability that Δ*β* > 0) for size further suggested the existence of missing traits or missing environmental factors, given that 94.2% of the posterior distribution of Δ*β*, was greater than zero (Figure 4). Therefore, Δ*β* provides substantial evidence that the potential discordance between the BE and STS predictions was caused by unmeasured traits/environments (regardless of the low power of our analyses). While female body size is likely to have causal effects on fitness components such as fecundity (de Eyto et al., 2015), the weight of the posterior distribution seems to indicate that indirect selection on unmeasured correlated traits, which could include the same trait(s) measured in males, may be constraining the evolution of larger body size. Our analysis of selection on body size in males suggested a lack of overall directional selection, which may weaken overall selection at a genetic level in females if body size is positively genetically correlated across the sexes, as might be expected. We attempted to explore this using a bivariate animal model of body size in males and females, but this model suffered from convergence issues. However, the caveat of low sample size constraining our ability to estimate directional selection on males must be borne in mind here.

One potential weakness of our study is the fact that our fitness measure, by necessity of sampling constraints, is an adult‐to‐adult measure of *LRS*. Evolutionary genetics theory traditionally asserts that fitness should be counted from conception to death, for example, the expected lifetime production of zygotes by a given zygote, thus avoiding complications associated with attributing offspring fitness components to parental fitness and conflating selection with inheritance (Cheverud, 1984; Grafen, 1988; Lande & Arnold, 1983). For example, selection pressures and evolutionary potential can be overestimated or underestimated when heritable maternal effects and their potential genetic covariance with direct genetic effects are not accounted for in a Breeder's Equation‐type analysis (Wolf & Wade, 2001; Wilson, Coltman, et al., 2005). In practical terms, adult‐to‐adult *LRS* measures are more easily obtained in salmonid populations (Reed et al., 2018; Seamons et al., 2007) than other types of individual‐level fitness measures such as adult‐to‐fry reproductive success, or egg‐to‐egg fitness, since assigning zygotes or juveniles to adults is made impractical by the sheer quantities of eggs/fry involved, and by their aquatic nature. Due to these difficulties in tracking individuals throughout their lifetime, our results must be considered in light of the “invisible fraction” sensu Grafen, (1988; Hadfield, 2008), which refers to situations where some individuals in the population die before a trait is measured or expressed and thus are “missing” in the accounting of overall selection pressures. For example, fast growth may be costly to survival, and thus, a part of the population that would otherwise express large adult body size could have died by the time adult body size is actually measured; hence, true selection on genes coding for larger fish may in fact be weaker. This is an issue faced by all (to the best of our knowledge) long‐term salmonid monitoring programs that typically are limited to sampling adults on their return to freshwater. While it may also be possible to monitor smolts on their migration from freshwater to saltwater, it may not be possible, or be otherwise unadvisable, to actually handle smolts at this vulnerable life‐stage, and in any case, it remains extremely difficult to get data on what happens to different phenotypes at sea. Thus, unless advances are made in our ability to track individual fish across their entire life (perhaps by the genetic tagging of fertilized ova in wild redds), the expansion of quantitative genetics and selection analyses in wild fish populations will remain somewhat hampered. Simulation studies could be used to better understand how the type of fitness measure influences evolutionary inferences under different scenarios of direct and indirect effects of trait on fitness.

### 4.4 Concluding remarks

Across three generations of our molecular pedigree, we could not demonstrate a clear pattern of change in body size for female Atlantic salmon at the phenotypic level, congruent with both the observed stasis in breeding values and the predicted evolutionary stasis according to the STS. We used the Δ*β* test to infer that missing traits correlated with female body size were likely present, and thus that using the estimated phenotypic selection gradient in the univariate BE will likely lead to a biased microevolutionary prediction—that is that larger body size should evolve, when in fact no evolutionary trend was observed, nor was any predicted by the STS. Our results caution against naïve expectations of directional evolution, even when the key “ingredients” of (apparent) directional selection and heritability are present, especially in studies where power may be low. By exploring evolutionary potential in a fish species, our study complements a growing literature reviewed by Pujol et al. (2018), most of which has been on birds and mammals, showing how mismatches between predicted and observed microevolution can result from a range of biological and statistical mechanisms. Additionally, we highlight how caution must be taken when interpreting results based on data‐poor systems with low power and limited sampling regimes. Many unanswered questions remain, however, such as the role of constraints due to sexual conflict and the stability of selection gradients and quantitative genetic parameters through time, or across age classes/environmental contexts, and whether a feasible solution to the invisible fraction problem will become available for highly fecund aquatic species such as Atlantic salmon. These issues are particularly important to understand/solve for body size and related traits in fish populations, given their key role in mediating eco‐evolutionary responses to anthropogenic changes (Naish & Hard, 2008), including climate change, harvest selection, and release or escape of captive‐reared fish into wild populations.

## CONFLICT OF INTEREST

The authors declare they have no conflicting interests with the work herein.

## AUTHOR CONTRIBUTIONS

RJOS, TER, and AK conceptualized the paper and designed the analyses, with RJOS conducting the analyses. PMcG, PAP, TA, SEJ, and CRP conceived the original Burrishoole pedigree construction project. PMcG, GR, and RP facilitated data collection and provided access to historical datasets. TA, SEJ, and CRP generated the molecular data and constructed the pedigree. RJOS, TER, and AK wrote the first draft of the manuscript, with all co‐authors contributing to subsequent drafts. TA wrote the text of Appendix 1 and produced Figures S2 and S3.

## Data Availability

Data and code used in the analyses are archived in the Marine Institute's online data repository at https://doi.org/10.20393/1b6fed63-4d4b-40f5-9473-32e8210e605a.

## APPENDIX 1

### Genotyping

In addition to the 29 microsatellite markers described in Aykanat et al., 2014, the MHCII region was used as a polymorphic marker (see Vähä, Erkinaro, Niemelä, & Primmer, 2007 for primer information), which was multiplexed together with “panel 2” markers (Aykanat et al., 2014).

### Sex determination

Both phenotypic sexing (i.e., identifying sex by expression of secondary sexual characteristics during sampling) and genetic assays were employed to determine the sex of an individual. The genetic sex determination is a PCR‐based presence and absence assay, which targets the sex determination gene, *sdy*, in Atlantic salmon (presence of *sdy* = male, absence of *sdy* = female; Yano et al., 2013, see Aykanat, Lindqvist, Pritchard, & Primmer, 2016 for primer information). The presence or absence of the *sdy* gene was determined by either agarose gel or using an ABI3130 fragment analyzer. In the agarose gel method, two laboratory workers independently evaluated the presence/absence of *sdy* amplification in the gel, using the 18S region as the positive control. In the fragment analyzer method, the normalized intensity of *sdy* amplification (log‐normalized to the intensity of the microsatellite markers, “ssosl438,” “ssa124,” “sssp1605,” “ssf43,” “ssa202,” and “sssp3016,” which were multiplexed with the *sdy* marker) was evaluated. The log‐normalized *sdy* intensity is expected to exhibit a clear bimodal distribution where females are expected to have zero, or close to zero normalized intensity values. As such, arbitrary thresholds of 0.05 and 0.13 were used as cut‐offs for sex determination (i.e., threshold <0.05 is female, and threshold >0.13 is male). Genetic sex determination was highly concordant within and between platforms. Concordance was 95.7% (44/46) among replicate assays between agarose and fragment analyzer methods. Likewise, sex determination within the fragment analyzer method was highly concordant with 98.6% (145/147) of individuals accurately sexed across replicated assays.

If genetic sex and phenotypic sex were in contradiction with each other, genetic sex was prioritized over phenotypic sex. Overall, phenotypic and genotypic sex were highly concordant within the final dataset, agreeing in 93.4% of cases (1,296/1,387).

### Identity analysis

Individual genotypes in the dataset may have identical or near‐identical genotypes due to contamination, or as a result of underlying biological reasons such as re‐sampling of the same fish within a given year, or across subsequent years (i.e., repeat spawning fish). To detect such genotypes, identity analysis was performed using Cervus 3.0.7, whereby an individual pair is considered as potentially identical, if they have a maximum of three mismatches, and the difference between matches and mismatches is at least 10. The resulting list of identical pairs were further inspected by eye and, based on the concordance of secondary information (length between putative identical pairs should be less than or equal to 4 cm and capture years should be biologically plausible), a decision was made and the pair were marked as biologically identical, or as a result of contamination throughout the processing of samples. The identity analysis was performed using a larger set of individuals which included cohorts from later years as well as fish with hatchery origins (*N* = 5,152). This gave a total of 13,268,976 pairwise genotype comparisons, of which 168 pairs matched the initial criteria (Figure S2). Most identical genotypes were biological (153/168), either being fish trapped both upstream and downstream (most of which were hatchery fish), or previous spawning fish which were sampled in subsequent years, and 15 were marked as contaminated. Finally, there were 34 “incidental” identical pairs that exhibited low mismatch (<4) but also low match‐mismatch difference (<10) due to low numbers of overlapping loci being successfully genotyped. One of these incidental pairs, that is, the one with lower genotyping success, was removed from downstream analyses. The identity analysis modestly altered the dataset used in the study: 32 pairs marked as previously spawned salmon were excluded from the dataset, and only two individuals in two incidental pairs were removed from further analysis. Only individuals genotyped at more than nine loci were included in the identity and the subsequent parentage analyses.

### Parentage analysis

A likelihood‐based parentage analysis was performed using Cervus 3.0.7. A combination of LOD scores (logarithm of the odds of an individual being a parent compared to the average likelihood score of the population) and delta scores (the difference in the likelihood scores of being the parent between the two most likely candidate parents) were used to assign parentage. A candidate parent was assigned to an offspring if the LOD score of a link was greater than the 95% LOD score threshold, or if the LOD score was between the 80% and 95% threshold score but with the delta score still higher than the 95% confidence threshold.

The cohort‐specific critical values for the log‐likelihood statistics for LOD scores and delta values were obtained using the simulation module in Cervus 3.0.7. For that, a conservative number of 1,000 parents for both mother and father, and 5,000 for offspring were simulated for each cohort—which are conservative estimates compared to census sizes for salmon from the Burrishoole system, or compared to unsampled parent estimates as in Aykanat et al. (2014). Cohort‐specific missing individual proportions and empirical missing genotype information were implemented in the simulations. Genotype error rates (ER1) were calculated the same as Aykanat et al. (2014) by averaging all values across loci, and between paternal and offspring cohorts. For cohorts that were not included in Aykanat et al. (2014), the ER1 was estimated using the linear regression formula that models ER1 as a function of time but restrained to a minimum value of 0.01. Allele frequency distributions were obtained using all wild samples from the system. Simulations were carried out for females and males separately. The paternity analysis was carried out, first using mothers only (maternity analysis), and then feeding confidently assigned mothers to the subsequent paternity analysis. Any resulting trios (both mother and father identified) were later inspected for the confidence of the likelihood father–mother–offspring link (i.e., trio confidence).

Our parentage analysis was also robust to false‐positive parentage assignments. We adapted an empirical test to quantify the rate of false discovery rates (FDR) in this study. For that, we performed another parentage analysis, identical to the aforementioned described analysis, but included parental candidates with zero probability of being first‐order relatives to the offspring cohort tested. These “improbable parent candidates” were from the Burrishoole population (either of wild or hatchery origin). Hence, we quantified the FDR by assessing the proportion of false positives (i.e., number of false‐positive links divided by number of links tested) to the proportion of parental links from probable cohorts. For example, in the 1977 cohort, 63 valid maternal parents were assigned out of 20,206 possible parent‐offspring combinations. Among impossible mother–offspring links, 63 were assigned out of 351,581 pairwise combinations tested. This provided an FDR of 0.0355778. Overall, the false discovery rate was small, with an average of 0.055 across cohorts (*SE* ± 0.025, see Table S2), further suggesting that the parentage testing was robust.
