# Supplementary figures and images for: Evolutionary stasis of a heritable morphological trait in a wild fish population despite apparent directional selection
Source: Ecol Evol. 2019 Jun 11;9(12):7096–111. doi: 10.1002/ece3.5274 (PMC6617767; doi:10.1002/ece3.5274)

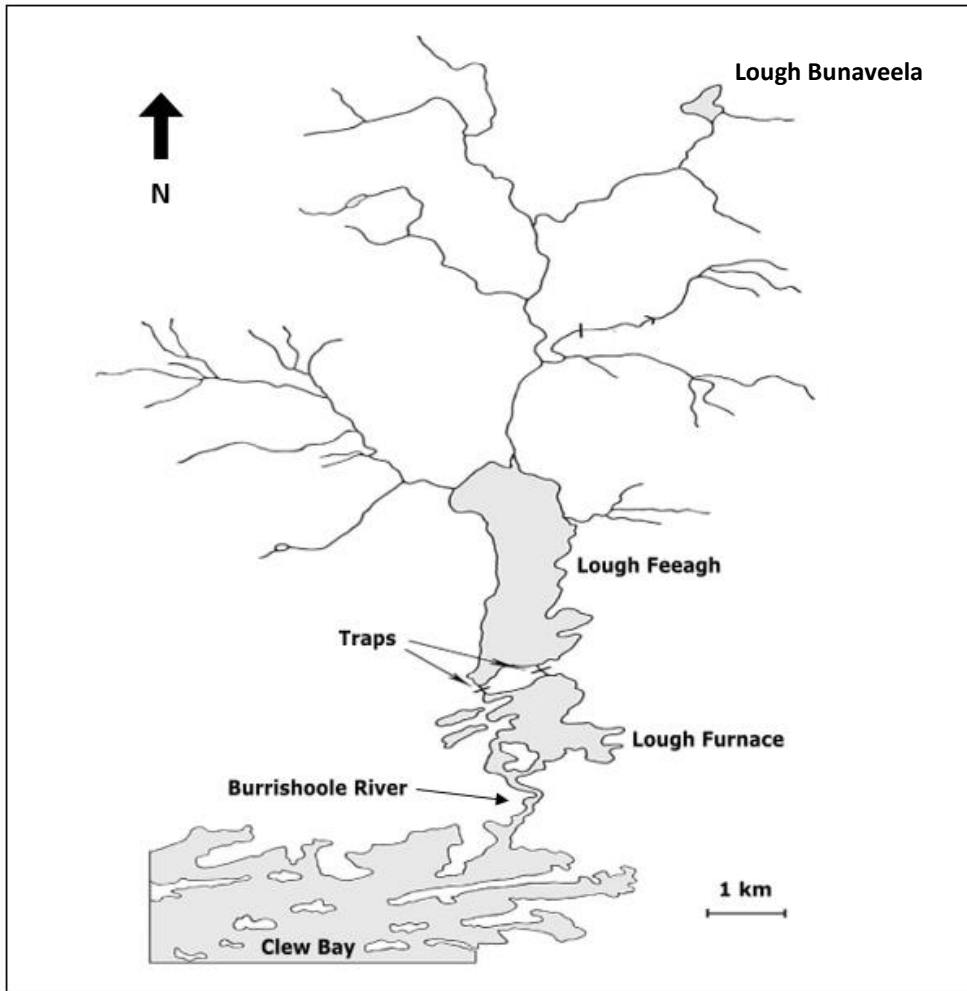

Figure S1: Map of the Burrishoole catchment, County Mayo, Ireland. Adapted from McGinnity et al., 2003.

Supplement: Supplementary file 1 [file ECE3-9-7096-s001.pdf]

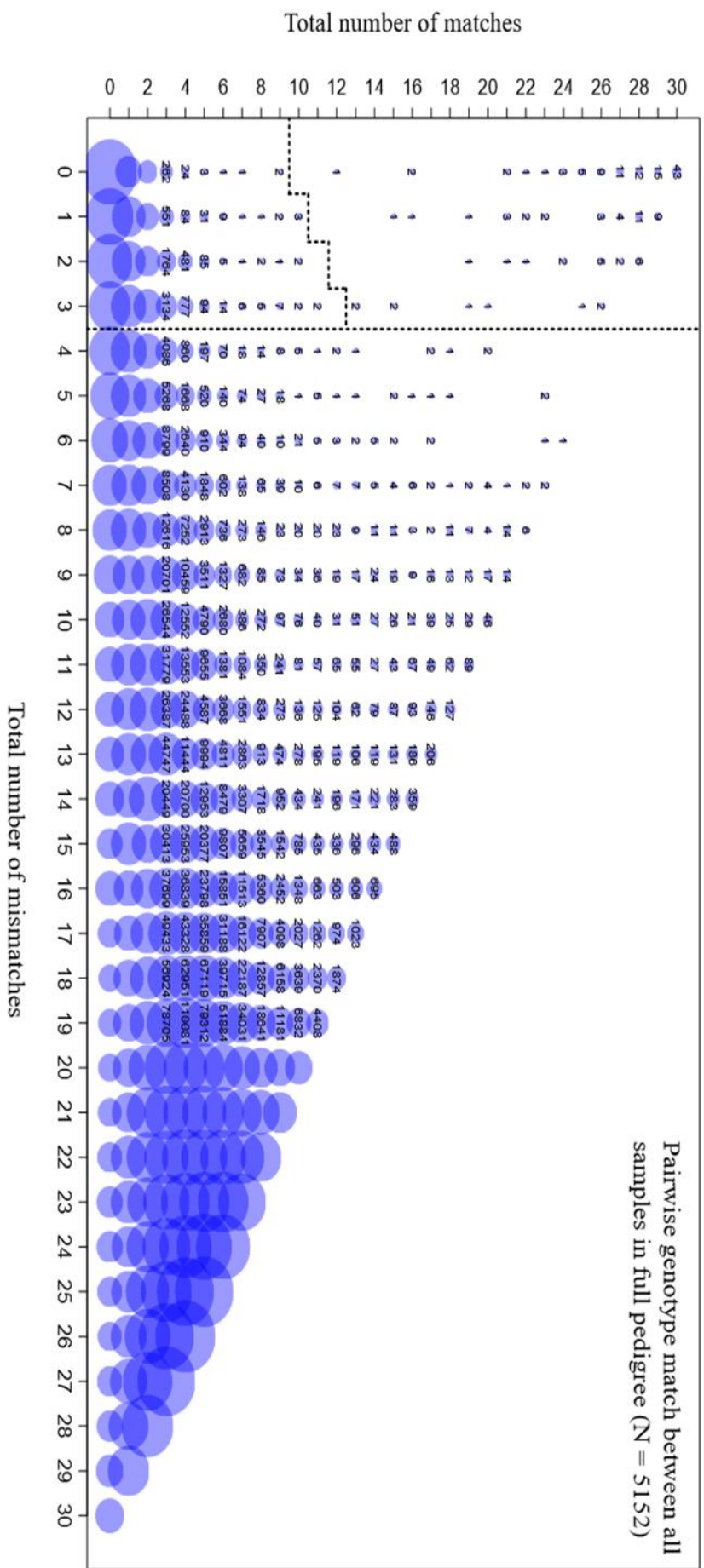

Supplement: Supplementary file 2 [file ECE3-9-7096-s002.pdf]
