# Supplementary material for: Evolutionary stasis of a heritable morphological trait in a wild fish population despite apparent directional selection
Source: Ecol Evol. 2019 Jun 11;9(12):7096–111. doi: 10.1002/ece3.5274 (PMC6617767; doi:10.1002/ece3.5274)
Supplement: Supplementary file 3 [file ECE3-9-7096-s003.pdf]

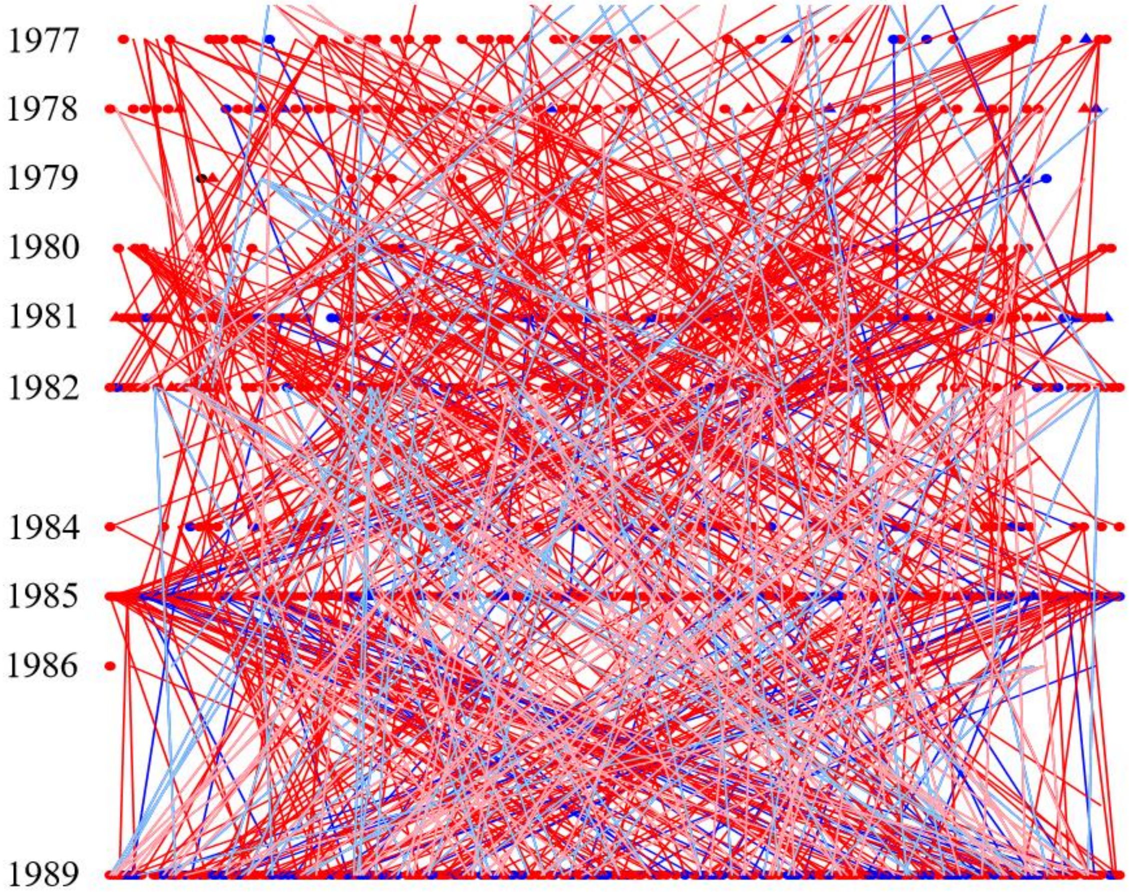

Figure S3: Pedigree of Atlantic salmon, *Salmo salar*, from the Burrishoole catchment, Ireland. Red lines represent maternities and blue lines represent paternities. Hatchery maternal/paternal links are represented with lighter shading. Hatchery and wild parents are represented with triangles and circles, respectively. Years on the y-axis represent the spawning cohort to which an individual belonged. The unrooted individuals at the top of the pedigree represent dams and sires whose offspring spawned in one of the study cohorts.
