# Supplementary material for: Evolutionary stasis of a heritable morphological trait in a wild fish population despite apparent directional selection
Source: Ecol Evol. 2019 Jun 11;9(12):7096–111. doi: 10.1002/ece3.5274 (PMC6617767; doi:10.1002/ece3.5274)
Supplement: Supplementary file 4 [file ECE3-9-7096-s004.pdf]

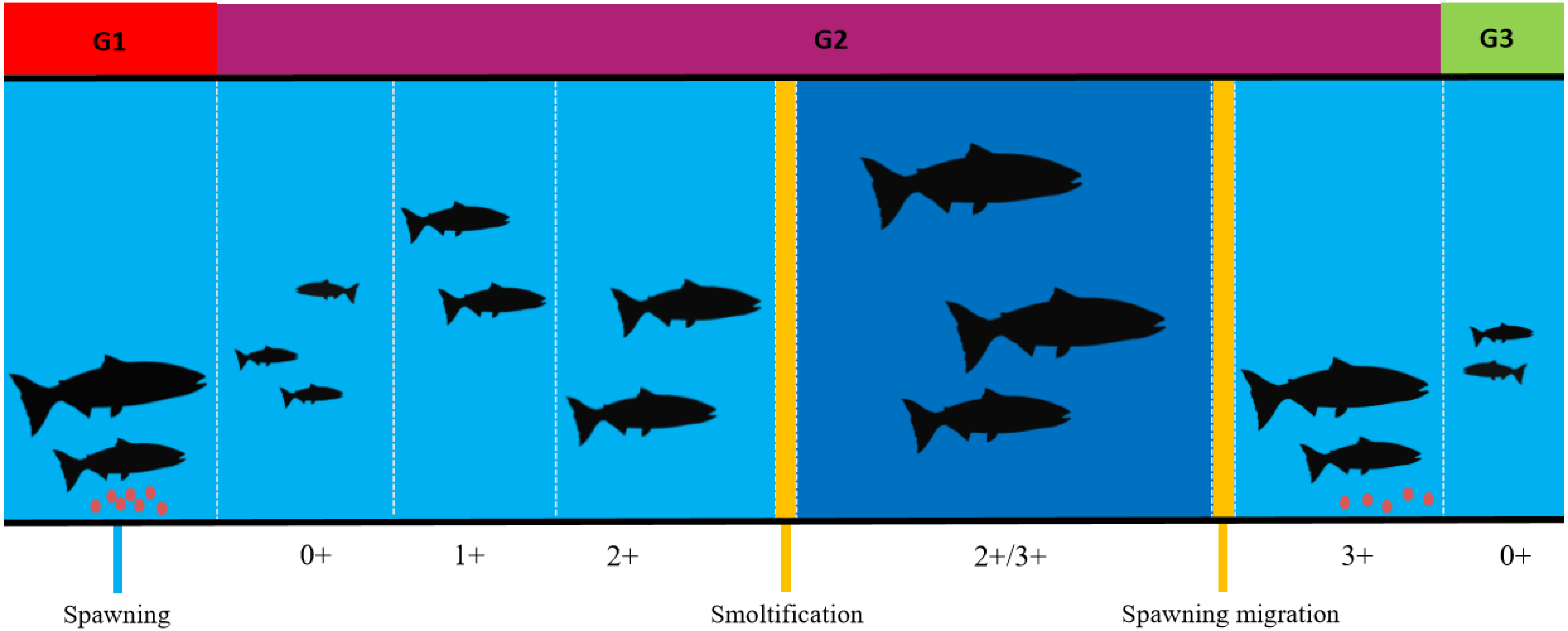

Figure S4: Stylized four-year lifecycle of Burrishoole Atlantic salmon. Adult fish spawn in freshwater, G1. G1's offspring, G2, typically spend 3 years in freshwater (0+, 1+, 2+) before leaving the river (smoltification) during their third year of life, and undergoing an oceanic feeding stage (2+/3+). G2 typically return to freshwater to spawn during their fourth year of life (3+), giving rise to the next generation, G3.
