# Supplementary material for: Evolutionary stasis of a heritable morphological trait in a wild fish population despite apparent directional selection
Source: Ecol Evol. 2019 Jun 11;9(12):7096–111. doi: 10.1002/ece3.5274 (PMC6617767; doi:10.1002/ece3.5274)
Supplement: Supplementary file 5 [file ECE3-9-7096-s005.docx]

| Table S1: The number of fish in the full pedigree (*Fish in full pedigree*) by year of spawning (*Cohort*). The pedigree is divided into the numbers of wild and hatchery fish sampled in a given cohort. The pedigree is further divided into female and male fish - sample size by sex (*No.fish*), mean length by sex (*Size*_mean_) with associated standard error (*Size*_SE_), and mean lifetime reproductive success by sex (*LRS*_mean_) with associated standard error (*LRS*_SE_). "-" denotes missing data. Missing data arose from no sampling of fish of a given provenance (wild or hatchery) in a given cohort, from no fitness being assigned to individuals in a given cohort, or from no sampling of a given sex in a given cohort. | | | | | | | | | | | | | | |
| --- | --- | --- | --- | --- | --- | --- | --- | --- | --- | --- | --- | --- | --- | --- |
|  |  |  |  |  |  |  |  |  |  |  |  |  |  |  |
|  |  |  |  |  |  |  |  |  |  |  |  |  |  |  |
|  |  |  |  |  |  |  |  |  |  |  |  |  |  |  |
|  |  |  |  | Female | | | | | | Male | | | | |
| Cohort | *Fish in each cohort of the full pedigree* | *Wild fish* | *Hatchery fish* | *No.fish* | *Size*_mean_ | *Size*_SE_ | *LRS*_mean_ | *LRS*_SE_ |  | *No.fish* | *Size*_mean_ | *Size*_SE_ | *LRS*_mean_ | *LRS*_SE_ |
| 1977 | 168 | 157 | 11 | 137 | 62.09 | ±0.40 | 0.5 | ±0.09 |  | 24 | 59.29 | ±4.90 | 0.42 | ±0.21 |
| 1978 | 147 | 83 | 64 | 102 | 64.58 | ±0.67 | 0.81 | ±0.15 |  | 33 | 64.97 | ±5.74 | 0.52 | ±0.21 |
| 1979 | 392† | 284 | 107 | 263 | 60.15 | ±0.23 | 0.48 | ±0.07 |  | 109 | 60.68 | ±10.44 | 0.39 | ±0.08 |
| 1980 | 154 | 137 | 17 | 118 | 61.34 | ±0.58 | 0.5 | ±0.08 |  | 13 | 61.37 | ±3.61 | 0.15 | ±0.10 |
| 1981 | 264 | 162 | 102 | 182 | 62.71 | ±0.51 | 0.66 | ±0.03 |  | 59 | 58.41 | ±7.68 | 0.53 | ±0.11 |
| 1982 | 237 | 164 | 73 | 174 | 60.77 | ±0.39 | 0.14 | - |  | 40 | 61.49 | ±6.32 | 0.13 | ±0.05 |
| 1983 | 291 | 230 | 61 | 218 | 62.09 | ±0.32 | - | ±0.06 |  | 38 | 61.61 | ±6.16 | - | - |
| 1984 | 164 | 131 | 33 | 91 | 59.66 | ±0.61 | 0.24 | ±0.05 |  | 46 | 57.46 | ±6.78 | 0.17 | ±0.06 |
| 1985 | 717† | 229 | 487 | 391 | 63.29 | ±0.22 | 0.54 | ±0.05 |  | 283 | 66.37 | ±16.82 | 0.37 | ±0.06 |
| 1986 | 591 | 53 | 538 | 326 | 63.55 | ±0.36 | - | - |  | 243 | 63.74 | ±15.59 | - | - |
| 1987 | 5 | 4 | 1 | 1 | 57.5 | - | - | - |  | 4 | 64.17 | ±2 | - | - |
| 1988 | 119 | 35 | 84 | 103 | 60.96 | ±0.74 | - | - |  | 15 | 55.5 | ±3.87 | - | - |
| 1989 | 775 | 334 | 441 | 465 | 61.77 | ±0.20 | 0.24 | ±0.03 |  | 297 | 61.79 | ±17.23 | 0.24 | ±0.04 |
| 1990 | 2 | - | 2 | 1 | 74 | - | - | - |  | 1 | 60 | ±1 | - | - |
| 1993 | 402 | 202 | 200 | 241 | 60.18 | ±0.35 | - | - |  | 157 | 60.38 | ±12.53 | - | - |
| 2001 | 47 | 47 | - | 42 | 60.76 | ±0.99 | - | - |  | 5 | 56.02 | ±2.24 | - | - |
| 2005 | 98 | 98 | - | 51 | 56.22 | ±0.42 | - | - |  | 43 | 52.98 | ±6.56 | - | - |
| 2008 | 1 | 1 | - | 1 | 63.8 | - | - | - |  | - | - | - | - | - |
| 2009 | 204 | 199 | 5 | 136 | 58.03 | ±0.54 | - | - |  | 45 | 59.11 | ±6.71 | - | - |
| Total | **3669^‡^** | **2550** | **2226** | **3043** |  |  |  |  |  | **1455** |  |  |  |  |
| "†" denotes a cohort where a single fish in the full pedigree was not assigned as either "wild" or "hatchery. “‡”: Not all fish could be assigned to a spawning cohort, hence the discrepancy between the total for column one and the sum of the totals for columns two and three. | | | | | | | | |  |  |  |  |  |  |
|  |  |  |  |  |  |  |  |  |  |  |  |  |  |  |
|  |  |  |  |  |  |  |  |  |  |  |  |  |  |  |

| Parentage | Parent cohort year | N _assigned,_  _probable links_ | N _total_  _probable links_ | P _probable_ | N _assigned,_  _improbable links_ | N _total_  _improbable links_ | P _improbable_ | FDR _weighted_ | FDR _cohort average_ |
| --- | --- | --- | --- | --- | --- | --- | --- | --- | --- |
| Females | 77 | 63 | 20206 | 0.003118 | 39 | 351581 | 0.000111 | 0.035578 |  |
|  | 78 | 94 | 19941 | 0.004714 | 34 | 423821 | 8.02E-05 | 0.017018 |  |
|  | 79 | 128 | 59772 | 0.002141 | 46 | 564110 | 8.15E-05 | 0.038079 |  |
|  | 80 | 59 | 17366 | 0.003397 | 34 | 334043 | 0.000102 | 0.029959 |  |
|  | 81 | 128 | 41804 | 0.003062 | 61 | 547844 | 0.000111 | 0.036365 |  |
|  | 82 | 21 | 9059 | 0.002318 | 20 | 123600 | 0.000162 | 0.069803 |  |
|  | 84 | 23 | 4773 | 0.004819 | 16 | 125326 | 0.000128 | 0.026494 |  |
|  | 85 | 208 | 132385 | 0.001571 | 70 | 768231 | 9.11E-05 | 0.057994 |  |
|  | 89 | 111 | 72200 | 0.001537 | 47 | 456109 | 0.000103 | 0.067026 |  |
|  | **Total** | 835 | 377506 | 0.002212 | 367 | 3694665 | 9.93E-05 | **0.044908** | **0.042035** |
|  |  |  |  |  |  |  |  |  |  |
| Males | 77 | 11 | 4145 | 0.002654 | 25 | 204565 | 0.000122 | 0.046051 |  |
|  | 78 | 10 | 6850 | 0.00146 | 29 | 241029 | 0.00012 | 0.082417 |  |
|  | 79 | 41 | 30667 | 0.001337 | 35 | 318468 | 0.00011 | 0.082203 |  |
|  | 80 | 2 | 2980 | 0.000671 | 36 | 180777 | 0.000199 | 0.296719 |  |
|  | 81 | 30 | 14368 | 0.002088 | 32 | 291369 | 0.00011 | 0.0526 |  |
|  | 82 | 2 | 2226 | 0.000898 | 16 | 66505 | 0.000241 | 0.267769 |  |
|  | 84 | 8 | 2277 | 0.003513 | 16 | 66296 | 0.000241 | 0.068692 |  |
|  | 85 | 101 | 99527 | 0.001015 | 51 | 358268 | 0.000142 | 0.140275 |  |
|  | 89 | 63 | 44880 | 0.001404 | 43 | 232504 | 0.000185 | 0.13175 |  |
|  | **Total** | 268 | 207920 | 0.001289 | 283 | 1959781 | 0.000144 | **0.112032** | **0.129831** |
|  |  |  |  |  |  |  |  |  |  |
| All | 77 | 74 | 24351 | 0.003039 | 64 | 556146 | 0.000115 | 0.037868 |  |
|  | 78 | 104 | 26791 | 0.003882 | 63 | 664850 | 9.48E-05 | 0.02441 |  |
|  | 79 | 169 | 90439 | 0.001869 | 81 | 882578 | 9.18E-05 | 0.049114 |  |
|  | 80 | 61 | 20346 | 0.002998 | 70 | 514820 | 0.000136 | 0.045352 |  |
|  | 81 | 158 | 56172 | 0.002813 | 93 | 839213 | 0.000111 | 0.039398 |  |
|  | 82 | 23 | 11285 | 0.002038 | 36 | 190105 | 0.000189 | 0.092914 |  |
|  | 84 | 31 | 7050 | 0.004397 | 32 | 191622 | 0.000167 | 0.037978 |  |
|  | 85 | 309 | 231912 | 0.001332 | 121 | 1126499 | 0.000107 | 0.080616 |  |
|  | 89 | 174 | 117080 | 0.001486 | 90 | 688613 | 0.000131 | 0.087943 |  |
|  | **Total** | 1103 | 585426 | 0.001884 | 650 | 5654446 | 0.000115 | **0.061013** | **0.055066** |
|  |  |  |  |  |  |  |  |  |  |

Table S2: Assessing the false discovery rate of parentage using empirically improbable parentage links as the source of false positive links for the full pedigree.

| Table S3: Number of wild fish enumerated in a given cohort (*Wild fish census*), sample size of fish used in these analyses (*No.fish*), mean length (*Size*_mean_) with associated standard error (*Size*_SE_), and mean lifetime reproductive success (*LRS*_mean_) with associated standard error (*LRS*_SE_) for female and male Atlantic salmon used in these analyses. Note that *Wild fish census* does not include hatchery fish that escaped up the catchment and spawned. | | | | | | | | | | | | |
| --- | --- | --- | --- | --- | --- | --- | --- | --- | --- | --- | --- | --- |
|  |  |  |  |  |  |  |  |  |  |  |  |  |
|  |  |  |  |  |  |  |  |  |  |  |  |  |
|  |  |  | Female | | | |  |  | Male | | | |
| Cohort | *Wild fish census* | *No.fish* | *Size*_mean_ | *Size*_SE_ | *LRS*_mean_ | *LRS*_SE_ |  | *No.fish* | *Size*_mean_ | *Size*_SE_ | *LRS*_mean_ | *LRS*_SE_ |
| 1977 | 594 | 128 | 62.29 | ±0.42 | 0.52 | ±0.1 |  | 20 | 59.25 | ±1.27 | 0.15 | ±0.08 |
| 1978 | 400 | 64 | 65.32 | ±0.96 | 1.19 | ±0.22 |  | 8 | 63.38 | ±2.96 | 0.38 | ±0.18 |
| 1979 | 854 | 208 | 60.23 | ±0.26 | 0.59 | ±0.08 |  | 63 | 58.87 | ±0.59 | 0.57 | ±0.13 |
| 1980 | 628 | 110 | 61.02 | ±0.61 | 0.47 | ±0.08 |  | 7 | 58.61 | ±1.40 | 0.29 | ±0.18 |
| 1981 | 355 | 124 | 62.73 | ±0.64 | 0.9 | ±0.11 |  | 22 | 58.4 | ±1.23 | 1.05 | ±0.24 |
| 1982 | 392 | 127 | 60.14 | ±0.43 | 0.17 | ±0.04 |  | 19 | 58.1 | ±0.89 | 0.16 | ±0.09 |
| 1984 | 345 | 81 | 59.31 | ±0.62 | 0.25 | ±0.06 |  | 30 | 56.5 | ±0.81 | 0.23 | ±0.08 |
| 1985 | 472 | 154 | 62.52 | ±0.36 | 1 | ±0.12 |  | 60 | 59.86 | ±0.58 | 0.92 | ±0.20 |
| 1989 | 501 | 189 | 61.02 | ±0.30 | 0.42 | ±0.1 |  | 73 | 60.36 | ±0.0.61 | 0.47 | ±0.1 |
| Total | **4541** | **1185** |  |  |  |  |  | **302** |  |  |  |  |
